# Editorial Comment: Misdiagnosis And Undertreatment Of Erectile Dysfunction In The Portuguese Primary Health Care

**DOI:** 10.1590/S1677-5538.IBJU.2020.06.06

**Published:** 2020-09-02

**Authors:** Valter Javaroni

**Affiliations:** 1 Departamento de Andrologia Hospital Federal do Andaraí Rio de Janeiro Rio de JaneiroRJ Brasil Departamento de Andrologia, Hospital Federal do Andaraí Rio de Janeiro, Rio de Janeiro, RJ, Brasil

Afonso Morgado ^1^, Maria Leonor Moura ^2^, Paulo Dinis ^3^, Carlos Martins Silva ^3^

^1^ Serviço de Urologia, Centro Hospitalar São João, Porto, Portugal; Departamento de Biomedicina, Faculdade de Medicina da Universidade do Porto, Porto, Portugal; ^2^ Faculdade de Medicina da Universidade do Porto, Porto, Portugal; ^3^ Serviço de Urologia, Centro Hospitalar São João, Porto, Portugal; Departamento de Cirurgia e Fisiologia, Faculdade de Medicina da Universidade do Porto, Porto, Portugal

Sex Med. 2019 Jun;7(2):177-183.

**DOI: 10.1016/j.esxm.2019.01.004 | ACCESS: 10.1016/j.esxm.2019.01.004**

## COMMENT

In this original research Dr. Afonso Morgado and cols. highlighted the need for continuous medical education on general practitioner (GP) level regarding sexual dysfunction in Portugal. What is the importance and role of the general practitioner in the management of erectile dysfunction (ED)?

It is important to remember that ED is a high prevalent disease and GP represent an important step in men’s health care. First, because ED is considered an independent cardiovascular risk marker ( 1 ). Second, because represents an opportunity to identify hidden risk factors and also to modify and improve daily habits ( 2 ). And third, because ED causes a huge suffering and loss of men´s quality of life. So it is important that GP could be able to identify properly and treat ED accordingly to best practice ( 3 ).

They assessed 200 men diagnosed by GP with Erectile Dysfunction (ED) that were refereed to specialized urology care. 115 were included. There was an important misdiagnosis of ED with 22.3% and the main reason was the presence of premature ejaculation. Only 33% had taken PDE 5 inhibitors. Less than 50% had been prescribed with the highest dose of PD5i. Almost 80% were considered with low cardiovascular risk.

Nowadays when the pharmaceutical industry is no more investing time and money to improve ED awareness among general practitioners, it is important that this gap could be fulfilled by non-profitable agencies/organizations to guarantee continuous medical education. Especially in undeveloped countries that do not have a health public system so organized as Portugal does.
